# Assessment of decorin-binding protein A to the infectivity of *Borrelia burgdorferi *in the murine models of needle and tick infection

**DOI:** 10.1186/1471-2180-8-82

**Published:** 2008-05-28

**Authors:** Jon S Blevins, Kayla E Hagman, Michael V Norgard

**Affiliations:** 1 Department of Microbiology, University of Texas Southwestern Medical Center, Dallas, Texas 75390, USA

## Abstract

**Background:**

Decorin-binding proteins (Dbps) A and B of *Borrelia burgdorferi*, the agent of Lyme disease, are surface-exposed lipoproteins that presumably bind to the extracellular matrix proteoglycan, decorin. *B. burgdorferi *infects various tissues including the bladder, heart, joints, skin and the central nervous system, and the ability of *B. burgdorferi *to bind decorin has been hypothesized to be important for this disseminatory pathogenic strategy.

**Results:**

To determine the role of DbpBA in the infectious lifecycle of *B. burgdorferi*, we created a DbpBA-deficient mutant of *B. burgdorferi *strain 297 and compared the infectious phenotype of the mutant to the wild-type strain in the experimental murine model of Lyme borreliosis. The mutant strain exhibited a 4-log decrease in infectivity, relative to the wild-type strain, when needle inoculated into mice. Upon complementation of the DbpBA-mutant strain with DbpA, the wild-type level of infectivity was restored. In addition, we demonstrated that the DbpBA-deficient mutant was able to colonize *Ixodes scapularis *larval ticks after feeding on infected mice and persist within the ticks during the molt to the nymphal state. Moreover, surprisingly, the DbpBA-mutant strain was capable of being transmitted to naïve mice via tick bite, giving rise to infected mice.

**Conclusion:**

These results suggest that DbpBA is not required for the natural tick-transmission process to mammals, despite inferences from needle-inoculation experiments implying a requirement for DbpBA during mammalian infection. The combined findings also send a cautionary note regarding how results from needle-inoculation experiments with mice should be interpreted.

## Background

The causative agent of Lyme disease, *Borrelia burgdorferi*, is introduced into a mammalian host via tick bite, whereupon the organisms enter the skin, disseminate hematogenously, and persist in the presence of a strong host immune response [1]. The dissemination and persistence of *B. burgdorferi *within a mammalian host is thought to be predicated, at least in part, on the organism's ability to bind molecules of the extracellular matrix (ECM) [2-4], inasmuch as these interactions have been shown to be important for other bacterial pathogens [5]. Among various ECM components, *B. burgdorferi *binds to type I collagen [4], fibronectin [6,7], integrins [8,9], the proteoglycan decorin [10], and glycosaminoglycans (GAGs) [11,12]. The *B. burgdorferi *proteins described as ECM-binding proteins include BBK32 [6,13], Bgp (*b*orrelia-*G*AG binding *p*rotein) [12], P66 (Oms66) [14], *d*ecorin-*b*inding *p*rotein (Dbp) A, and DbpB [10,15]. These proteins, and perhaps other as yet unidentified molecules, may play a significant role in the infectivity and pathogenesis phenotypes of *B. burgdorferi*.

Our laboratory has been interested in DbpA as a vaccine candidate for the prevention of Lyme disease and the contribution of DbpA and DbpB to *B. burgdorferi *pathogenesis and infectivity [16,17]. Since their *in vitro *characterization as decorin-binding proteins [10,15], the DbpA and DbpB lipoproteins have been implicated as potential contributors to adhesion and colonization of *B. burgdorferi *within mammalian hosts [15,18]. The genes that encode DbpA and DbpB reside in an operon, *dbpBA*, on linear plasmid 54 (lp54) and are found within many *B. burgdorferi sensu lato *isolates [19]. Neither protein is expressed by *B. burgdorferi *within the tick vector [17], however, expression of *dbpA *(and presumably *dbpB*) is upregulated in the mammalian host after ticks deposit spirochetes into the skin [20,21]. DbpA and DbpB expression likely remains high for the duration of mammalian infection, as inferred by the presence of antibodies against both antigens in the serum of mice as late as one year after infection (Hagman, unpublished data). The presence of antibodies against DbpA in the serum of patients with late-stage, disseminated Lyme disease also is well-documented [22], providing added support for DbpA expression by *B. burgdorferi *during chronic infection.

Although the combined data to date suggest an important role for the decorin-binding proteins of *B. burgdorferi *during mammalian infectivity, virtually all data inferring the importance of DbpA and DbpB thus far have been indirect. The first direct investigation into the role of the *dbpBA *operon in the infectious lifecycle of *B. burgdorferi *was carried out by Shi et al [23]. In this study, mutational analysis of *dbpBA *in *B. burgdorferi *strain B31 indicated that neither DbpA, nor DbpB was essential in the murine needle-challenge infection model of borreliosis. However, there was evidence suggesting that these mutants exhibited a modest level of attenuation in immunocompetent mice. Unfortunately, a comprehensive 50% infectious dose (ID_50_) was not included in this report to further investigate this possible defect, nor was genetic complementation of the mutation performed. This latter point is of particular importance given the genetic plasticity of *B. burgdorferi *and the possibility that spontaneous loss of an endogenous borrelial plasmid might account for the apparent defect in this mutant. To more directly examine the role of both DbpA and DbpB in the murine/tick model of Lyme disease, a *dbpBA*-deficient mutant and a *dbpA *genetic complement of the mutant were generated in the infectious strain 297 of *B. burgdorferi*. Phenotypic assessment of mouse needle infectivity by ID_50 _analysis and *Ixodes scapularis *tick colonization and tick-transmission capacity by the Bb297 *dbpBA*-mutant also were performed.

## Results

### Construction and characterization of a *dbpBA*-deficient mutant

**Figure 1:**
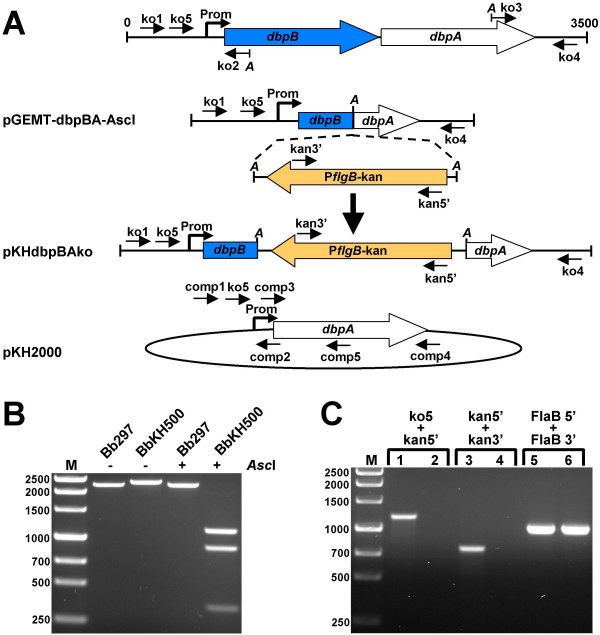
**Construction of *dbpBA*-deletion mutant BbKH500 and Prom-*dbpA *complementation vector and PCR confirmation**. (A) Strategy for the replacement of the *dbpBA *operon with the P*flgB*-kan and complementation with pKH2000. pKHdbpBAko was the pGEM-T easy-based suicide plasmid used to transform Bb297 for the homologous recombination of the kanamycin-resistance gene into the *dbpBA *operon. "A" denotes *Asc*I sites. P*flgB*-kan denotes the kanamycin-resistance marker expressed from the *flgB *promoter. The borrelial shuttle vector containing the *dbpBA *Prom fused to the *dbpA *ORF (pKH2000) was transformed into BbKH500 to restore DbpA expression. Oligonucleotide primers used for PCR are indicated with short arrows. (B) PCR using primers ko5 and ko4 (shown in panel A). The first two lanes are undigested PCR products from Bb297 and BbKH500, whereas the second two lanes are the corresponding PCR products digested with *Asc*I. (C) Lanes 1, 3 and 5 are PCR products derived from BbKH500 template DNA and lanes 2, 4 and 6 are PCR products derived from Bb297 template DNA. Primer pairs used in PCR are indicated above the lanes. FlaB5' and FlaB3' primers amplify *flaB *of *B. burgdorferi*. DNA size standards (M) are shown in base pairs on the left.

To assess the roles that DbpA or DbpB play in the infectivity and pathogenesis of *B. burgdorferi*, we created a *dbpBA*-deficient mutant by allelic exchange of a P*flgB*-kan cassette for the majority of the *dbpBA *operon (Fig. 1A). Infectious Bb297 was chosen for construction of the *dbpBA *mutant because it is a human isolate [24] and prior infectious mutants of this strain have been readily created [25-28].

Bb297 was electroporated with the suicide vector, pKHdbpAko, containing a P*flgB*-kan cassette flanked by 1.5 kb of DNA on the left side of the *dbpBA *operon and 890 bp of DNA on the right side of the *dbpBA *operon (Fig. 1A). This allelic exchange strategy relies on a double crossover, homologous recombination event to replace the *dbpBA *operon with the P*flgB*-kan cassette. Several kanamycin-resistant transformants were obtained, and the presence (Fig. 1B) and orientation (Fig. 1C) of the P*flgB*-kan cassette within the *dbpBA *operon was confirmed by PCR analysis.

**Figure 2:**
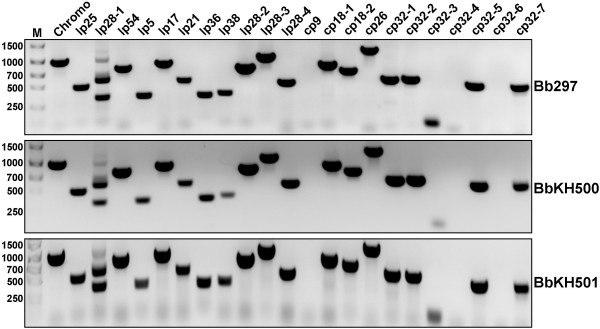
**PCR-based plasmid profiling to compare the plasmid contents of the strains employed in this study**. PCR amplification with primers specific for each of the known endogenous *B. burgdorferi *plasmids was used to compare the plasmid content of parent Bb297, BbKH500, and BbKH501. Sequence information for the primers utilized is provided in Table 4. Plasmid designations above each lane are based on strain B31 plasmid annotation. DNA size standards (M) are shown in base pairs.

**Figure 3:**
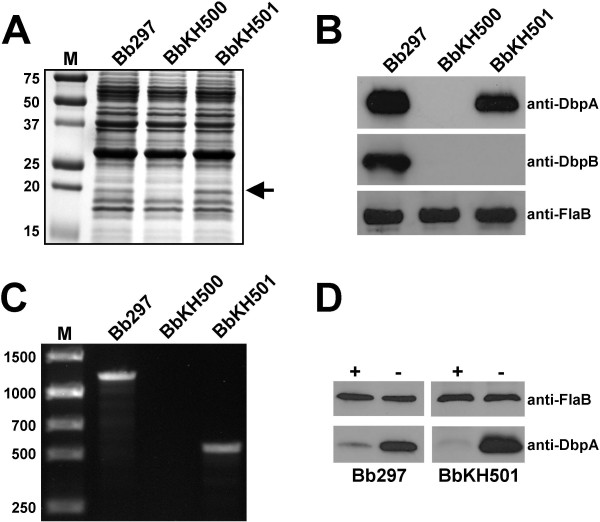
**Characterization of BbKH500 and BbKH501**. (A) Whole-cell lysates of Bb297, BbKH500, and BbKH501 were resolved by SDS-PAGE (12.5% acrylamide) and stained with Coomassie brilliant blue or (B) or transferred to nitrocellulose and assessed by immunoblot analysis with antibodies as noted on the right. (C) PCR analysis of Bb297, BbKH500 and BbKH501 for presence of *dbpA*. (D) Immunoblot analysis of whole-cell lysates of proteinase K-digested (+) or undigested (-) Bb297 or BbKH501. Antibodies used noted on the right. Molecular mass of markers (M) in panel A are shown in kDa. The arrow at the right in panel A denotes the protein band corresponding to DbpA. DNA size standards (M) in panel C are shown in base pairs.

During genetic manipulation and *in vitro *cultivation, *B. burgdorferi *may spontaneously lose endogenous plasmids that are not required for growth *in vitro*, but are essential for mammalian infection. At least two plasmid-encoded genes, *vlsE *[29] and *pncA *(BBE22) [30], fit this criteria. One kanamycin-resistant transformant, BbKH500, retained both *vlsE *and *pncA *(Fig. 2) and was analyzed by immunoblotting to confirm the loss of DbpA and DbpB. As expected, DbpA and DbpB were absent from BbKH500 (Figs. 3A and 3B). BbKH500 exhibited identical doubling times when compared to wild-type Bb297 (data not shown) and PCR-based plasmid profiling revealed that the endogenous plasmid profile of BbKH500 matched that of the parent Bb297 (Fig. 2).

### Loss of DbpBA significantly reduces the infectivity of *B. burgdorferi *in mice when introduced via needle inoculation

To determine the contribution of DbpA and DbpB to the infectivity of *B. burgdorferi *in mice, C3H/HeJ mice were challenged intradermally via needle inoculation with increasing numbers of BbKH500 (10^4^, 10^5^, 10^6 ^and 10^7 ^spirochetes). As a positive control, an additional five mice were inoculated with wild-type Bb297 at a dose of 10^3 ^spirochetes (ID_50 _of approximately 50 spirochetes; [27]). The results are shown in Table 1. Whereas the mice infected with 10^3 ^of Bb297 showed culture positive ear punch biopsies at two weeks post-infection, needle challenge of naïve mice with 10^4 ^BbKH500 did not produce an infection in any mouse (n = 15), even when the infection was allowed to progress for 14 weeks. Seventeen mice challenged with 10^5 ^and ten mice challenged with 10^6 ^BbKH500 also did not show signs of infection (negative ear-punch biopsy culture) at four weeks post-challenge (Table 1). However, after 10 weeks, three mice from the group infected with 10^5 ^BbKH500 and two mice from the group challenged with 10^6 ^BbKH500 were shown to be infected by positive ear-punch biopsy cultures. Additionally, mice were challenged with either 10^5 ^or 10^6 ^BbKH500 and ear-punch biopsies were harvested 14 weeks after infection (Table 1). From the group that received 10^5 ^spirochetes, two mice were infected (n = 10), and from the group that received 10^6^spirochetes, four mice were infected (n = 10). All of the mice challenged with 10^7 ^of BbKH500 had positive ear-punch cultures as early as five-weeks post-challenge, and as late as 14 weeks post-infection (n = 5). These spirochetes from ear punch-positive cultures were analyzed by diagnostic PCR as described above, and confirmed to be Bb297 or BbKH500 (data not shown). Aliquots of these cultures also were passed to media containing kanamycin to confirm resistance and sensitivity of BbKH500 and Bb297, respectively. Seroconversion analyses performed on a subset of mice also revealed that none of the mice infected with BbKH500 showed antibody reactivity against DbpA or DbpB (data not shown). Furthermore, only mice that exhibited culture-positive ear punch cultures showed significant serum antibody reactivity with P39 (data not shown). Analysis of all infection results yielded an ID_50 _for BbKH500 of 1.2 × 10^6 ^(p <0.001) compared with ID_50 _of approximately 50 bacteria for wild-type Bb297 [27].

**Table 1:** Assessing infectivity of BbKH500 in needle-challenged C3H/HeJ mice.

	Inoculation dose (bacteria/inoculation)			
	
Time post-infection (wks)	10^4a^	10^5a^	10^6a^	10^7a^
5	0/10	0/17	0/10	5/5
10	ND	3/17	2/10	ND
14^b^	0/5	2/10	4/10	5/5
Cumulative results	0/15	5/27	6/20	10/10

^a ^Mice were needle infected with varying concentrations of bacteria. Infection rates were assessed at the noted timepoints by culturing BbKH500 from ear-punch biopsies.

^b ^Mice from 14 week timepoint represent groups distinct from the 5- and 10-week groups.

### Complementation of the *dbpBA*-deletion mutant with DbpA restores infectivity in needle-challenged mice

The results above suggested that the *dbpBA *operon is required for full infectivity of *B. burgdorferi *when mice are infected via needle inoculation. However, during genetic manipulation of *B. burgdorferi*, it is not uncommon to lose one or more plasmid(s) which potentially contribute to infection, therefore genetic complementation is necessary to definitely ascribe the attenuated phenotype in BbKH500 to the *dbpBA *lesion. Although BbKH500 carries a mutation in both DbpA and DbpB, we chose to complement with only *dbpA *because i) DbpA is better characterized that DbpB [31,32] and ii) experimentation suggests that DbpA is the prominent Dbp in *B. burgdorferi *[15,33]. To restore DbpA expression in BbKH500, a shuttle plasmid carrying a copy of *dbpA *driven by its native promoter was constructed (Fig. 1A). Since *dbpA *is the second gene in the *dbpBA *operon, we chose to clone the promoter for this operon upstream of the *dbpA *gene and then insert the fusion product in the multiple-cloning site of pJD51. BbKH500 cells were transformed with this shuttle plasmid (pKH2000), yielding clones resistance to both kanamycin and streptomycin. These transformants were screened by PCR to verify the presence of the complementing plasmid (Fig. 3C). Clones shown to contain the complementing plasmid were checked for *vlsE *and *pncA *(Fig 2.), as described above. PCR-based plasmid profiling, performed on one of the transformants that contained both *pncA *and *vlsE*, revealed a profile identical to the parent strain, BbKH500 (Fig. 2). This clone, designated BbKH501, was selected for further analysis and DbpA expression was confirmed by both SDS-PAGE (Fig. 3A) and immunoblot analysis (Fig. 3B). Proteinase K digestion of BbKH501 and Bb297, under conditions that left FlaB intact, demonstrated surface exposure of DbpA in both strains (Fig. 3D). Three groups of five C3H/HeJ mice were needle inoculated (i.d.) with BbKH501 at 10^2^, 10^3 ^or 10^4 ^borreliae per mouse. Ear punch biopsies were harvested at either two weeks (10^3 ^and 10^4 ^doses) or three weeks (10^2 ^dose); all 15 mice became infected, as confirmed by positive ear-punch biopsy cultures. Although a precise determination of the ID_50 _of BbKH501 could not be calculated, the fact the 100% of the mice were infected with 10^2 ^bacteria suggests that the ID_50 _is less than 10^2 ^spirochetes (4-logs lower than BbKH500). Aliquots of these cultures were passed to media containing streptomycin and kanamycin selection to confirm resistance. Diagnostic PCR also confirmed that the spirochetes growing out of the ear punch biopsy were BbKH501 (data not shown). Seroconversion analyses carried out on a subset of mice also revealed that the mice infected with BbKH501 showed antibody reactivity against DbpA, but not DbpB (data not shown). These data confirm that DbpA is necessary for wild-type levels of *B. burgdorferi *infectivity in mice when introduced via needle inoculation, and that DbpB is dispensable for this aspect of the infectious process.

### Neither DbpA nor DbpB is required for acquisition of *B. burgdorferi *by ticks or infection of mice via tick bite

To determine whether *I. scapularis *ticks could acquire borreliae lacking *dbpA *and *dbpB*, naïve larvae were allowed to feed to repletion on mice infected with BbKH500 or on mice infected with Bb297. Larvae were collected after feeding, allowed to molt, and the unfed nymphs were examined for the presence of *B. burgdorferi *by IFA; approximately 11 weeks had elapsed since these ticks had fed to repletion as larvae. Microscopic examination of multiple fields revealed approximately one spirochete per field in the BbKH500-infected ticks (23 ticks examined), as opposed to 20–30 spirochetes per field in the Bb297-infected ticks (10 ticks examined). All Bb297-infected ticks examined were positive for *B. burgdorferi*. Of the ticks that had fed on BbKH500-infected mice, 80% were positive for spirochetes.

Because the ticks maintained strain BbKH500 through their molt, it was important to assess whether the loss of DbpA and DbpB affected *B. burgdorferi *transmission to mice via tick bite. To test this, five BbKH500-infected nymphs were placed on each of two naïve mice and ten BbKH500-infected nymphs were placed on each of six naïve mice. An additional two naïve mice received five Bb297-infected ticks each. The ticks were allowed to feed to repletion, and at three weeks post-infestation, ear-punch biopsies were harvested from all mice. The ear-punch biopsies were cultured in BSK medium and the cultures examined by dark-field microscopy for the presence of *B. burgdorferi*. These results are shown in Table 2. One of the two mice infested with five ticks harboring BbKH500 was infected as demonstrated by a positive ear-punch culture, and four of the six mice infested with ten ticks harboring BbKH500 were infected. Both of the mice on which the Bb297-infected ticks fed had positive ear-punch biopsy cultures. These spirochetes were analyzed by diagnostic PCR as described above, and confirmed to be Bb297 or BbKH500 (data not shown). Seroconversion analyses performed on a subset of the BbKH500-infected mice also revealed that none of the mice infected with BbKH500 showed antibody reactivity against DbpA or DbpB (data not shown). Furthermore, only mice that exhibited culture-positive ear punch cultures showed significant antibody reactivity against P39 (data not shown). Taken together, these data indicate that DbpA and DbpB are not essential for the transmission of *B. burgdorferi *from ticks to mice.

**Table 2:** Assessing infectivity of BbKH500 via tick inoculation of C3H/HeJ mice

Strain	Ticks/mouse^a^	Mouse infectivity^b^
Bb297	5	2/2
BbKH500	5	1/2
	10	4/6

^a ^Varying number of ticks infected with BbKH500 were allowed to feed to repletion on naïve mice.

^b ^Infection rates were assessed at three weeks post-infestation by culturing BbKH500 from ear-punch biopsies

## Discussion

The importance of ECM-binding proteins to the pathogenic strategy of *B. burgdorferi *is not currently known. It is presumed that borreliae bind mammalian host cells during infection because binding to various ECM molecules and to tissue culture cells has been demonstrated *in vitro *[4,34,35]. Of the known ECM-binding proteins of *B. burgdorferi*, Bgp [36] and BBK32 [37] have been deleted from infectious strains N40 and B31, respectively, and shown to be dispensable for infectivity in the mouse model of Lyme disease. A deletion of integrin-binding protein, P66, in a noninfectious strain of *B. burgdorferi*, HB19 [38], caused the spirochetes to lose their ability to bind b_3_-chain integrins [14] but the phenotype of a P66 mutant in an infectious strain of *B. burgdorferi *is currently unknown. The remaining two known ECM-binding proteins, DbpA and DbpB, were the focus of this work due to multiple lines of evidence suggesting a role for one or both proteins during infection of mammalian hosts [15,18,31]. To directly address the contribution of both DbpA and DbpB to *B. burgdorferi *infectivity, we created BbKH500, a *dbpBA*-deletion mutant of the human isolate Bb297, and used this strain to challenge mice with increasing numbers of spirochetes. We observed a 4-log reduction in infectivity of the mutant strain when the spirochetes were needle inoculated into mice. Complementation of the *dbpBA*-mutant with DbpA alone in the *dbpBA*-mutant restored infectivity of *B. burgdorferi *to wild-type levels.

Prior to this work, Fischer et al. [18] had restored DbpA and DbpB expression (via shuttle plasmid) to *B. burgdorferi *strain B314, a non-adherent, noninfectious derivative of strain B31 that has lost several plasmids, including lp54 [39]. Although expression of the Dbp molecules restored binding of the spirochetes to purified decorin, dermatan sulphate, and human epithelial cells [18], experiments to test the infectivity of B314 expressing DbpA and DbpB in the murine model of Lyme disease were implausible due to the loss of plasmids that are required for mammalian infection [39]. A recent study by Shi et al. [23] assessed the infectivity of a DbpA,B mutant of *B. burgdorferi *strain 5A18NP1, a B31 derivative that lacks the BBE02 gene [40]. Based on needle-challenge results obtained from a single inoculation dose of 10^5 ^bacteria (80% infection), Shi et al. [23] ascertained that the *dbpBA *locus was not required for infectivity. However, in the present study, we observed a 4-log increase in the ID_50 _of BbKH500 (>10^6 ^bacteria) and a 19% infection rate with a dose of 10^5 ^BbHK500 when mice were infected by needle inoculation. The reason for the difference in infectivity levels of the *dbpBA*-deficient mutants reported by Shi et al. and BbKH500 created in our laboratory is unknown at this time. One possible explanation for this disparity could be due to our use of Bb297 as the parental strain for the present mutational analysis, whereas Shi et al. [23] utilized a clonal derivative of B31 that is a BBE02-mutant. This variability also could be explained by spirochete enumeration differences prior to needle challenge. As mentioned in the Methods, significant attention was given to the enumeration of BbKH500 prior to needle inoculation of mice.

It should be noted that during the course of the study described herein, a subsequent report on the role of DbpBA was published by Shi et al. [41], which, unlike their previous study, included genetic complementation experiments. Our current findings agree with those of this most recent report in that Shi et al. also observed a 4-log increase in ID_50 _values with their *dbpBA *mutant [41]. In addition, Shi et al. observed a defect in the ability of the *dbpBA *mutant to colonize heart, joint and skin tissues, suggesting an overall deficiency in dissemination [41]. The observation that mice infected with BbKH500 showed a delay in infectivity (10- to 14-weeks post-infection) also suggests that BbKH500 might be attenuated with respect to its capacity to disseminate through the host. However, whereas we were able to compensate fully for the loss infectivity in the *dbpBA *mutant by complementing with *dbpA *alone (as assessed by ear punch biopsy culture), Shi et al. reported that both *dbpA *and *dbpB *were required to restore the infectivity of their *dbpBA *mutant [41]. Although the precise reason for this disparity is unknown at this time, there are several differences between the experimental approaches of these studies that might account for these differing results. First, it is possible that some intrinsic differences(s) in the bacterial strains utilized might account for the variation observed between these two studies. Specifically, as noted above, strain 297 was the parental strain used in the current study, and Shi et al. utilized a highly-transformable clone (5A13) of strain B31 that lacks both lp56 and the virulence-associated plasmid lp25 as the background for their mutagenesis experiments [30,41]. Second, the disparity in our results might be due to the use of different strains of mice in these two studies; C3H/HeJ mice were used in our study, whereas Shi et al used BALB/c [41]. This may be relevant because numerous studies have reported that experimental infection of C3H strains of mice with *B. burgdorferi *results in significantly different disease pathologies [42,43], higher spirochetal loads in multiple tissues [44], and different cytokine responses [45,46] by comparison to similarly infected BALB/c mice. Although, it is difficult to precisely predict how the reported differences in pathogenesis observed in these two distinct murine backgrounds might be impacted by DbpA and/or DbpB during infection, it is reasonable to suspect that these differences might account for some of the disparity in the results of complementation experiments obtained in the aforementioned studies.

Even though DbpA was required for full infectivity by Bb297 in mice when introduced by needle inoculation (intradermally), neither DbpA, nor DbpB, was required for infection of larval *I. scapularis *ticks or for transmission of BbKH500 from infected ticks to naïve mice. That DbpA was not required for uptake of the spirochetes by larval ticks was not surprising due to our earlier work demonstrating that DbpA is not expressed by *B. burgdorferi *harbored within the tick vector [17]. However, based on the data we obtained from needle inoculation of mice with BbKH500, it was surprising that as few as five nymphal ticks could transmit BbKH500. This is especially surprising because semi-quantitative analysis of the total number of spirochetes observed in the midguts of unfed ticks infected with BbKH500 showed a lower spirochete density (approx. 1/field) by comparison to number of bacteria present in the dissected midguts of the Bb297-infected ticks (20–30/field). While these data might suggest a possible defect in either the efficiency of acquisition of the *dbpBA *mutant or the capacity of this mutant to persist in the tick midgut, there were still sufficient numbers of the mutant spirochetes in these ticks to infect naïve mice. Since it is not known precisely how many spirochetes are transmitted by ticks during the feeding process [47], it was impossible to directly compare the number of *B. burgdorferi *transmitted by tick bite to our needle-challenge experiments. Spirochete numbers within salivary glands during tick feeding have been estimated between 20 [47] and 61 spirochetes [48] per salivary gland pair. These data suggest that an individual tick deposits far fewer than 10^4 ^spirochetes during the feeding process, and our results have shown that by the needle-inoculation route, 10^4 ^BbKH500 are not infectious.

In addition to the data presented by Fischer et al. [18], additional reports have attempted to address the role of DbpA, DbpB, and the ECM proteoglycan, decorin, with respect to the infectivity and pathogenesis of *B. burgdorferi*. Brown et al. described *B. burgdorferi *infection of decorin-deficient mice (Dcn^-/-^) [49]. Brown et al. [49] found that, by comparison to Dcn^+/+ ^mice, Dcn^-/- ^mice challenged with a higher dose of *B. burgdorferi *(10^4^) had i) fewer infected joints, ii) a reduction in the severity of arthritis, but iii) no significant defect in colonization of the other tissues. Whereas 10^4 ^wild-type *B. burgdorferi *were infectious for Dcn^-/- ^mice in the studies by Brown et al. [49], the Bb297 *dbpBA*-mutant was unable to infect mice at this dose. When considered together, these data support the hypothesis that DbpA, in addition to binding decorin, may have an additional ligand(s) or has another function critical for infectivity. At the present time, the only other known ligand recognized by DbpA is the GAG dermatan sulfate [18], but the contribution of this interaction to the pathogenesis of *B. burgdorferi *remains to be elucidated.

## Conclusion

Despite the disparities between the results of the complementation experiments described in the current study and those obtained by Shi et al. [41], the overall results of our needle-inoculation experiments are in agreement with the most recent conclusion of Shi et al. that mutation of *dbpBA *results in significant attenuation of *B. burgdorferi *in the murine model of Lyme borreliosis. However, the observation that the *dbpBA *mutant showed a significant reduction in infectivity when the mice were needle inoculated is overshadowed by the finding that this same mutant was capable of infecting mice via tick challenge. The fact that DbpA and DbpB are dispensable for infection via the tick-mediated route of infection suggests that *B. burgdorferi *transmitted via tick bite are in some way phenotypically different than their *in vitro*-cultivated counterparts, and/or that tick-derived salivary components, such as Salp15, may assist *B. burgdorferi *during the early infection process [50]. Taken together, these results emphasize the importance of characterizing the impact of a given gene in the infectious lifecycle of *B. burgdorferi *using the natural tick vector, as opposed to using only the artificial needle-challenge model.

## Methods

### Bacterial strains and growth conditions

Infectious, low-passage *B. burgdorferi *strain 297 (Bb297) [51] was used for these studies. Bacteria were cultivated *in vitro *in either Barbour-Stoenner-Kelley (BSK)-II [52] or BSK-H Incomplete medium (Sigma-Aldrich, St. Louis, MO) supplemented with 6% normal rabbit serum (Pel-Freeze Biologicals, Rogers, AR) at 35°C with 5% CO_2_. When necessary, BSK media was supplemented with borrelia antibiotic mix (BAM; Sigma-Aldrich), 600 μg/ml kanamycin, or 700 μg/ml streptomycin.

### Generation of DbpBA deletion mutant in Bb297

The *dbpBA*-deficient Bb297 strain was created by allelic exchange of the *dbpBA *operon with a kanamycin-resistance cassette, P*flgB*-kan [53], derived from pBSV2 [54]. The mutagenesis construct, pKHdbpBAko, was created by generating two PCR products that constituted the left and right flanking regions of the *dbpBA *operon which then were joined via an *Asc*I restriction site, thus deleting a significant portion of the *dbpBA *operon. Takara EX Taq polymerase (Takara Bio Inc., Shiga, Japan) and oligonucleotide primers ko1 and ko2 (Table 3) were used to amplify the left arm and ko3 and ko4 (Table 3) were used to amplify the right arm; primers ko2 and ko3 were modified such that *Asc*I restriction sites would be introduced into the "middle termini" of the two PCR fragments. The resulting PCR products then were digested with *Asc*I and ligated together. The linear ligated product was used as the template in a second PCR amplification containing the primers ko1 and ko4. The resulting PCR product, representing the joined flanking regions, was cloned into pGEM-T Easy vector (Promega Corp., Madison, WI) to generate pGEMT-dbpBA-AscI. The P*flgB*-kan cassette was from pJD55, a derivative of pJD44 in which the original *aph *[3']*-IIIa *was replaced with the P*flgB*-kan cassette of pBSV2 [27,54]. In pJD55, the P*flgB*-kan cassette is flanked by *Asc*I sites which facilitated the cloning of the marker into the unique *Asc*I site within pGEMT-dbpBA-AscI to create pKHdbpBAko. Primers kan5' and kan3' were used with primers ko1 and ko4 to determine the orientation of the P*flgB*-kan cassette with respect to the *dbpBA *operon.

**Table 3:** Oligonucleotide primers used for cloning and PCR confirmation.

Designation	Sequence
ko1	GGATCTTAAGAATTTCAAATTTT
ko2	TATAGGCGCGCCAATACTACATGCGACCAAT^a^
ko3	TATAGGCGCGCCTGAAGAGAATCCTCCAACT^a^
ko4	TTTAGATTCTAAAGTTTAGATAAAAATTGGTCGGG
ko5	AAACAAGTCTTAAAATCACAAGC
kan5'	AGCCATATTCAACGGGAAACG
kan3'	TTCATATCAGGATTATCAATACC
	
vlsE-5'	GATGCAGAGAAGGCTGCTGCTGCAGTTAGTGC
vlsE-3'	TATAAGCTTTCATCAGAGAGTCTTATTAACAGCAGTCTCAAC
BBE22-5'	AAATTAATTTCTTTGATCAACCAAC
BBE22-3'	TATATTAAGCTTACTTTGGCTGTCG
FlaB5'	ATGATTATCAATCATAATACATCAGCTATTAA
FlaB3'	TTATCTAAGCAATGACAAAACATATTGGGGAA
	
comp1	GGCTTCTCTTTTATTTTTAAGACC
comp2	CATATGTTCCTCCTTCTATTAAATTTAGTTAAATTTAAATTTTAGCCCAC^b^
comp3	AGATCTCATATGATTAAATGTAATAATAAAACTTT^b^
comp4	GCATGCCTTTGGGTTAATTGCTTTAAC^c^
comp5	GTAGCTCCACTTTTGCTTC

^a ^*Asc*I site underlined.

^b ^*Nde*I site underlined.

^c ^*Sph*I site underlined

Bb297 were made electrocompetent and transformed with pKHdbpBAko as described by Yang et al [28]. After electroporation with pKHdbpBAko, spirochetes were allowed to recover overnight at 35°C without antibiotic selection in 20 ml BSK-H. The cells were diluted in BSK-H medium containing the appropriate concentration of antibiotic(s) and aliquoted in 96-well tissue culture plates (Corning, Lowell, MA). Transformants were recovered 7–21 days after plating from wells in which a red to orange color change of the medium was observed. The presence of viable spirochetes was confirmed visually by dark-field microscopy and clones expanded into BSK medium supplemented with kanamycin.

Transformants were verified as *dbpBA*-deficient mutants by diagnostic PCR using primers ko5 and ko4 (Table 3) followed by analytical restriction enzyme digestion of the PCR product with *Asc*I. DNA for PCR analysis was extracted from borreliae harvested from the expansion cultures. The PCR product generated from amplification of Bb297 DNA (wild-type) is of similar size to that generated by amplification of DNA from a *dbpBA*::P*flgB*-kan mutant, but the former lacks *Asc*I sites. Therefore, to verify that the *dbpBA *operon was replaced by the P*flgB*-kan cassette, PCR products from both Bb297 and mutant-derived were digested with *Asc*I prior to agarose-gel electrophoresis. The presence of the *vlsE *and *pncA *(BBE22) genes in kanamycin-resistant transformants was confirmed by PCR using vlsE-5' and vlsE-3' for *vlsE *amplification or BBE22-5' and BBE22-3' for BBE22 amplification; refer to Table 3 for sequence information. FlaB5' and FlaB3' primers, which amplified the *flaB *gene of *B. burgdorferi*, were used as a control for DNA integrity. A single *dbpBA*-deficient clone, BbKH500, that retained *vlsE *and *pncA*, was chosen for additional PCR-based analyses to compare the endogenous plasmid content of this clone to Bb297. The sequences of the primers utilized for plasmid profiling are provided in Table 4. Nine of the primer pairs have been previously described by Eggers et al. [55]. The remaining primers utilized are unique from those cited by Eggers et al. and were designed primarily based on sequence data from strain 297.

**Table 4:** Oligonucleotide primers used for plasmid profiling.

Primer	Sequence	Reference
lp54 5'	ATGAGCAAAAAAGTAATTTTAATAT	[55]
lp54 3'	CACTAATTCTTTTTGAATTACTAAT	[55]
cp26 5'	ATGCCTCCAAAAGTGAAGATAAAAA	[55]
cp26 3'	TAGCTTATAATTAAAAATTATTGAT	[55]
cp9 5'	ATGCAAAAAATAAACATAGCTAAAT	[55]
cp9 3'	ATCTTCTTCAAGATATTTTATTATA	[55]
lp17 5'	GTGTATACTGACCCAAGGTCAATTA	[55]
lp17 3'	CAATAATGTGATATTTTTAAGAAAT	[55]
lp25 5'	AAATTAATTTCTTTGATCAACCAAC	This study
lp25 3'	TATATTAAGCTTACTTTGGCTGTCG	This study
lp28-1 5'	GATGCAGAGAAGGCTGCTGCTGCAGTTAGTGC	This study
lp28-1 3'	TATAAGCTTTCATCAGAGAGTCTTATTAACAGCAGTCTCAAC	This study
lp28-2 5'	ATGGCGCTGATTACATTAATTGTCG	[55]
lp28-2 3'	AATCTTGAAGAACCTTGCATCTTTA	[55]
lp28-3 5'	CTGAAAATGAAGGAGAAGCGGGTGG	[55]
lp28-3 3'	TAGGCTAATACCAATTCGTACAAAT	[55]
lp28-4 5'	ATGAAATGCCATATAATTGCAACTA	[55]
lp28-4 3'	AATCCGACAGATCTGGTTTGTCCAG	[55]
lp38 5'	ATGATTATTACCCAAACAACGCCC	This study
lp38 3'	TTTTAAATCCATTTTCACAATATG	This study
lp36 5'	TTCTTATCCCTGACTTTCACTTTTGAGG	This study
lp36 3'	TCCTTTACTTCTATGTTTTTACTTTCCTTGGT	This study
lp5 5'	ATGAATGGAATAATTAACGATACAC	[55]
lp5 3'	AATATTAGGATGAAGATTATAAATT	[55]
lp21 5'	TGTGGTTGCTAAAACCCAAGCGT	This study
lp21 3'	TTGTTTCTAATTGCTCTGAATTGCATCC	This study
Chrom 5'	GATTATCAATCATAATACATCAGC	[55]
Chrom 3'	TCTAAGCAATGACAAACATATTGG	[55]
cp18-1 5'	AGGGGAATGTATTAATTGATAATTCA	This study
cp18-1 3'	AGATTTTTTCAAAACATTTGGCGAT	This study
cp18-2 5'	TCAGAAAGCATACCATTACAAGACAAC	This study
cp18-2 3'	AATAATACCTTTTTCTACGCCCGATA	This study
cp32-1 5'	GTTATAATACCTATTCAAGCAGAAAGG	This study
cp32-1 3'	GCTCCCTTCTAATACTTTTCTATAA	This study
cp32-2 5'	CAAGCGAGTTTATTCCCCTTAAA	This study
cp32-2 3'	ATTCTAATATTGTCCACTTTATGAAAT	This study
cp32-3 5'	ACTTGCAAGAGCACAGGTCTATAATTA	This study
cp32-3 3'	CTTAATACAATTAACGTTTCCAGTATA	This study
cp32-4 5'	GTATAAATGCTTTTGGTTATAAGCACAC	This study
cp32-4 3'	GAAACTCCTTCTCTAACCTTTACATAC	This study
cp32-5 5'	GCCTTATAAGGAACATAGGTTAAAGG	This study
cp32-5 3'	AGATTTCAAGCGCTCCTTCAACAAA	This study
cp32-6 5'	GGTGCTTTAGACACAAGAGATGTG	This study
cp32-6 3'	GAACAAATTTCAGATTTAACATTTATCG	This study
cp32-7 5'	GTCAAATTTAAGCTGTTTTAGCAGTG	This study
cp32-7 3'	TATTTACTAATCTATTTTTCAATTTTTCA	This study

### Construction of shuttle plasmids for genetic complementation of BbKH500 with DbpA

Complementation of DbpA in BbKH500 was achieved by transforming electrocompetent BbKH500 with the *B. burgdorferi*-shuttle plasmid, pKH2000. Because *dbpB *precedes *dbpA *in the native operon, it first was necessary to clone the promoter for the *dbpBA *operon directly in front of the *dbpA *gene from Bb297, thereby removing the *dbpB *gene. The *dbpBA *promoter region (Prom) was amplified by PCR using primers comp1 and comp2 and the *dbpA *open reading frame (ORF) was amplified using primer comp3 and comp4. To facilitate fusion of the Prom and *dbpA *open reading frame fragment, an *Nde*I restriction site was introduced into the 3' end of oligonucleotide comp2 and the 5' end of primer comp3. Following PCR amplification, the resulting PCR fragments were digested with *Nde*I and ligated together. A second PCR amplification was performed using the ligation product as the template and the oligonucleotides comp1 and comp4 for primers. The resultant PCR product was digested with *Bgl*II (a unique *Bgl*II site is located in the PCR product, 11 bp downstream of the 3' end of the comp1 primer) and *Sph*I (5' end of comp4 primer) then cloned into the *Bgl*II and *Sph*I sites of pJD51, a derivative of pJD44 [27], that contains the *aadA *gene encoding streptomycin resistance in *B. burgdorferi *[56], to create pKH2000. Electroporation of BbKH500 was performed as described above and transformants were selected in the presence of kanamycin and streptomycin. Antibiotic-resistant clones first were checked for the presence of the Prom-*dbpA *construct by PCR using primers ko5 and comp5 (the latter anneals near the middle of the *dbpA *coding strand); Bb297 = 1.2 kb product; BbKH500 = no product; Prom-*dbpA *= 535 bp product. Next, DbpA expression in clones identified by PCR confirmation was assessed by immunoblot analysis as described below. Clones that expressed DbpA from the Prom-*dbpA *construct were assessed for the presence of the *vlsE *and BBE22 genes by PCR amplification using the primers described above. One clone, BbKH501, was chosen for further characterization. PCR-based plasmid profiling was performed on BbKH501, as described above, to compare the plasmid content of this clone to BbKH500 and Bb297.

### Proteinase K digestion of *B. burgdorferi*

Intact, motile borreliae were exposed to 200 μg of proteinase K (40 mg/ml; Fisher Scientific, Pittsburgh, PA) or were sham treated for 40 min at room temperature. To stop the reaction, 10 μl of phenylmethylsulfonyl fluoride (50 mg/ml in isopropanol; Sigma) was added to each sample and the bacteria prepared for SDS-polyacrylamide gel electrophoresis (PAGE) and immunoblot analysis as described below.

### Immunoblot analysis

*B. burgdorferi *whole-cell lysates were generated by washing the spirochetes with wash buffer (10 mM HEPES, 150 mM NaCl, pH 7.5) three times, incubating the cells in BugBuster plus Benzonase solution (Novagen, Madison, WI) overnight then adding an equal volume of 2× SDS-PAGE running buffer (Bio-Rad Laboratories, Hercules, CA) for a final concentration of 10^7 ^bacteria/ml. Whole-cell lysates were separated via electrophoresis through 12.5% SDS-polyacrylamide gels (approximately 10^7 ^spirochetes per lane) and transferred to nitrocellulose (0.45 μm; Bio-Rad Laboratories) for immunoblot analysis. Nitrocellulose membranes were probed with either 6B3-DbpA, a mouse monoclonal antibody that specifically recognizes DbpA, chicken anti-*B. burgdorferi *FlaB IgY, or rat anti-DbpB anti-sera. The monoclonal antibody, 6B3-DbpA, was produced in collaboration with the Antibody Production Core facility at UT Southwestern and the polyclonal rat anti-DbpB antisera was described previously [16]. The chicken anti-FlaB antibody was produced in collaboration with Lampire Biological Laboratories (Pipersville, PA). Secondary antibodies were horseradish peroxidase-conjugated goat anti-mouse immunoglobulin G (IgG), donkey anti-chicken IgY, or goat anti-rat IgG (Jackson ImmunoResearch Laboratories, Inc., West Grove, PA) diluted 1:10,000–1:30,000 for chemiluminescent detection. Immunoblots were developed using Immobilon Chemiluminescent Western HRP Substrate (Millipore, Billerica, MA) and exposed to X-ray film (Phenix Research Products, Hayward, CA).

### Infection of mice by needle inoculation

Prior to use of the cultures in needle-inoculation experiments, the bacterial cell density in each culture was accurately determined by counting spirochetes in no fewer than 60 microscopic fields (400× magnification) using dark-field microscopy. Cultures exhibiting cell aggregation were not used for infections as the presence of clumps prevented accurate enumeration of spirochete density. Cultures with spirochetes exhibiting reduced motility also were not used for mouse infections. Three- to five-week-old female C3H/HeJ mice (The Jackson Laboratory, Bar Harbor, ME) were used for all studies. Mice were infected via intradermal injection with serial dilutions of *B. burgdorferi *in BSK medium as previously described [16]. At the appropriate time intervals, ear-punch biopsies were harvested using a 2 mm ear punch, placed in BSK-H medium supplemented with BAM, and cultures were examined by dark-field microscopy for the presence of spirochetes. Aliquots of each culture were passed to media containing antibiotics to confirm the antibiotic-resistance phenotype of the bacteria that were recovered from cultures of the ear punch biopsies. The ID_50 _was calculated using the method described by Reed and Muench [57]. UT Southwestern is accredited by the International Association for Assessment and Accreditation of Laboratory Animals Care (AAALAC) and all animal protocols were approved by the Institutional Animal Care and Use Committee (IACUC) at UT Southwestern.

### Colonization of *Ixodes scapularis *larvae with *B. burgdorferi*

Female C3H/HeJ mice were needle inoculated as described above with either Bb297 (10^4 ^spirochetes) or BbKH500 (100 μl of a post-exponential growth phase culture; 10^6^-10^7 ^spirochetes). Infection of mice was confirmed by ear-punch biopsy culture; 4- and 8-weeks post-inoculation for Bb297 and BbKH500, respectively. Naïve, pathogen-free *I. scapularis *larvae, obtained from the Department of Entomology and Plant Pathology at Oklahoma State University (Stillwater, OK), were allowed to feed to repletion on the infected mice individually housed in cages with raised wire-bottoms above water to facilitate recovery of the ticks. Fed larvae were collected and washed sequentially with 70% ethanol, deionized water (dH_2_O), 1× Fungizone (Gemini Bio-Products, West Sacramento, CA), and dH_2_O before placing them in 100% cotton fabric-lined Petri dish molting chambers for storage until they molted to the nymphal stage. The molting chambers containing the ticks were housed in a humidified chamber (97–98% humidity) containing saturated potassium sulfate solution at 20°C with a 16 h light, 8 h dark cycle. Unfed nymphs were collected and stored in autoclaved glass vials containing approximately 1 cm of sand. The vials were closed with vented lids and the ticks housed as described above.

### Direct immunofluorescence assay (IFA) on *B. burgdorferi*-infected ticks

Prior to placement of unfed (flat) nymphs on naïve mice, five nymphs from each of two individual mice were dissected on silylated slides (CEL Associates, Inc., Pearland, TX) in 50 μl phosphate-buffered saline containing 10 mM MgCl_2_. At the time testing was performed, approximately 11 weeks had elapsed since these ticks had fed to repletion as larvae. Midgut tissues were extracted from the ticks, allowed to dry on the slides, blocked with Tris-buffered saline with 0.1% Tween-20, then probed with a FITC-conjugated rabbit anti-*B. burgdorferi *antibody (Fitzgerald Industries International, Inc., Concord, MA) as described previously [27].

### Transmission of *B. burgdorferi *from infected nymphal ticks to naïve mice

To assess the transmissibility of strain Bb297 and BbKH500 from flat nymphs to naïve mice, either five or ten ticks were allowed to feed to repletion on three- to five-week-old female C3H/HeJ mice. At three-weeks post-infestation, ear-punch biopsies were harvested from each mouse and cultured in BSK-H medium without antibiotics to determine infection status. Borreliae from the ear-punch biopsy cultures were transferred to BSK-H medium supplemented with kanamycin and streptomycin to confirm their antibiotic-resistance phenotypes.

## Statistical analysis

Statistical analysis was performed with assistance from the UT Southwestern Clinical Sciences Department. Both chi-square and Fisher's exact test were applied to the mouse infection data in pair-wise comparisons between experimental groups.

## Authors' contributions

JSB and KEH performed experiments and analyzed results. JSB, KEH, and MVN participated in experimental designs and co-wrote the manuscript. All authors read and approved the manuscript.
